# Advancing High-Performance Metal Matrix Composites: Uniting Nature’s Design and Engineering Innovation

**DOI:** 10.3390/ma16186077

**Published:** 2023-09-05

**Authors:** Behzad Sadeghi

**Affiliations:** Department of Innovation Engineering, University of Salento, Via per Arnesano, 73100 Lecce, Italy; b.sadeghi2020@gmail.com

We are pleased to present this Special Issue entitled “Advanced High-Performance Metal Matrix Composites (MMCs),” which explores promising materials science that will change everything from aerospace to automotive technology. Through this collection of research contributions, we aim to explore the fascinating synergy between nature’s engineering principles and the innovative possibilities of technology. Inspired by playful biological materials such as shell nacre, this Special Issue combines the wonders of natural architectures with cutting-edge technology of human creation.

The main theme of this Special Issue revolves around the search for high-performance metal matrix composites, with a focus on aluminium-based variants. Addressing a critical challenge for the aerospace and automotive industries [1,2], our authors explore the delicate balance between the strength and ductility of these composites [3,4]. Since materials with improved mechanical properties are in demand in these areas, our first objective is to manufacture and develop composite materials that precisely meet this need. By tailoring architectural designs, we aim to overcome the traditional trade-off between strength and ductility and usher in a new era of engineering materials [5,6,7].

A salient feature of this Special Issue is the exploration of heterostructure strategies to strengthen metal matrix composites using advanced powder metallurgy techniques. By harnessing the power of powder metallurgy [8], our researchers are unlocking ways to develop particulate metal matrix composites with exceptional strength and ductility. This intricate interplay of materials and processes opens doors to novel applications and unparalleled performance capabilities.

We are also involved in the field of intelligent powder processing, an area in which technology meets innovation [9]. Our employees are actively working to develop engineered particles with tailored properties that breathe life into the vision of high-strength, lightweight metal matrix composites. These include breakthrough advances such as core–shell structures, advanced powder materials with deactivated sintering properties and revolutionary improvements in maintaining the discharge capacity of lithium-ion batteries. Of particular note in this Special Issue is the mastery of uniform dispersion and structural integrity, especially in the area of nano-carbon reinforcements such as CNTs and graphene.

Lastly, we turn our attention to the dynamic properties of metal matrix composites throughout their life cycle and uncover widely applicable property relationships [10,11]. By closely examining core–shell powders produced through intelligent dry processing, we are finding new avenues to improve performance and functionality.

In conclusion, we would like to thank the authors, reviewers and contributors who made this Special Issue possible. Their dedication and insights have paved the way for advances that can transform the industry and improve the fabric of modern engineering. We invite you, our valued readers, to dive into this collection and join us in celebrating the convergence of nature’s wisdom and human innovation.

## Data Availability

Not applicable.
